# Efficient and stable single-doped white OLEDs using a palladium-based phosphorescent excimer†
†Electronic supplementary information (ESI) available. See DOI: 10.1039/c7sc02512b


**DOI:** 10.1039/c7sc02512b

**Published:** 2017-09-11

**Authors:** Tyler Fleetham, Yunlong Ji, Liang Huang, Trenten S. Fleetham, Jian Li

**Affiliations:** a Materials Science and Engineering , Arizona State University , Tempe , AZ 85287 , USA . Email: jian.li.1@asu.edu

## Abstract

A tetradentate Pd(ii) complex, Pd3O3, which exhibits highly efficient excimer emission is synthesized and characterized. Pd3O3 can achieve blue emission despite using phenyl-pyridine emissive ligands which have been a mainstay of stable green and red phosphorescent emitter designs, making Pd3O3 a good candidate for stable blue or white OLEDs. Pd3O3 exhibits strong and efficient phosphorescent excimer emission expanding the excimer based white OLEDs beyond the sole class of Pt complexes. Devices of Pd3O3 demonstrate peak external quantum efficiencies as high as 24.2% and power efficiencies of 67.9 Lm per W for warm white devices. Furthermore, Pd3O3 devices in a carefully designed stable structure achieved a device operational lifetime of nearly 3000 h at 1000 cd m^–2^ without any outcoupling enhancement while simultaneously achieving peak external quantum efficiencies of 27.3% and power efficiencies over 81 Lm per W.

## Abstract 2

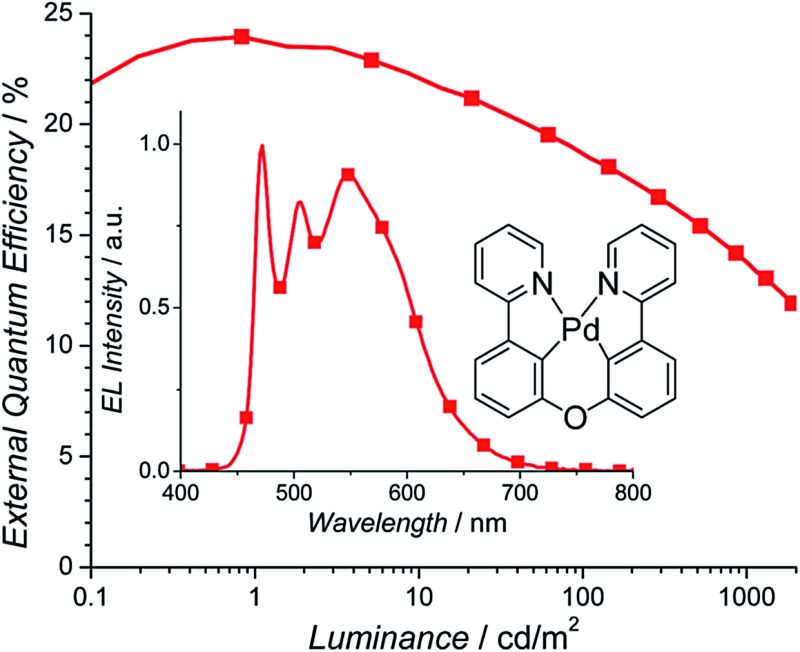
An efficient and stable palladium based excimer emitter was developed capable of exhibiting maximum EQE over 27% and achieved nearly 3000 h at 1000 cd m^–2^.

## 

White organic light emitting diodes (WOLEDs) are considered as strong candidates for next generation illumination devices due to their high efficiencies exceeding 100 Lm per W, potential for low cost and scalable production, and compatibility with flexible substrates.^1^–^3^ Through continuous improvements in device designs and by employing phosphorescent Ir or Pt emitters, WOLEDs with high efficiencies and high color quality have been achieved.^4^–^13^ Moreover, the imaginative curved, flexible and efficient OLED panels can assist in revolutionary luminaire design beyond serving the benefit of energy efficiency improvement. However, to prevent “color aging” and enhance the color stability, the state-of-the-art WOLED structures typically adopt multiple-stacked (tandem) OLEDs, resulting in a high manufacturing cost. Thus, improvement in device performance is not sufficient to realize the commercialization of WOLED and the reduction of device complexity for lower manufacturing costs is necessary.

Phosphorescent excimer-based WOLEDs are one of the most promising solutions to significantly lower the fabrication cost of WOLEDs.^14^–^18^ Single doped WOLEDs of this type decrease the complexity of device fabrication, provide a greater control of emission color and eliminate color aging due to the incorporation of single emissive materials. The recent works carried in our groups and others have demonstrated that single-doped WOLEDs can realize 100% internal electron-to-photon conversion efficiency in device settings and produce a high quality of white light.^16^–^18^ However, excimer-based devices face the same operational lifetime challenges as the conventional white OLEDs due to the lack of stable and efficient blue phosphorescent emitters. While many efficient blue emitters have been developed, very few have shown promising operational stabilities.^19^–^26^ The origin of the low operational stability of existing blue phosphorescent materials is unresolved and is under intensive investigation.^27^–^29^ The choice of cyclometalating ligands may be one of the major factors leading to the poor operational stability. Many of the most commonly used efficient blue emitters typically contain fluorinated cyclometalating ligands which have been shown to undergo fluorine loss.^30^,^31^ Furthermore, a recent report demonstrated that blue emitters containing phenyl-azole ligands exhibited much shorter device operational lifetimes than emitters of the same emission energy and similar energy levels which do not contain any five-membered heterocycles.^32^ Thus, it would be desirable to design blue phosphorescent emitters that employ the cyclometalating ligands similar to those of reported stable green and red phosphorescent emitters (*i.e.* the absence of fluorine groups or 5-membered heterocycles).

Stable and efficient single-doped WOLEDs require the development of phosphorescent materials with sufficient blue emission, efficient excimer emission, and a molecular design aligned with known stable phosphorescent emitters. However, Ir and Pt complexes have been unable to satisfy this desirable combination of characteristics, necessitating a new avenue of materials development. Pd(ii) complexes also have the potential for efficient excimer based white emission due to their square planar molecular geometry.^33^–^35^ However, Pd(ii) complexes have received significantly less attention than their Ir and Pt analogs. This is partially because Pd complexes have typically been non-emissive or weakly emissive due to their low radiative decay rates and low lying metal-centered (MC) states providing non-radiative decay pathways.^36^,^37^ Furthermore, none of the previous reports of Pd emitters, to our knowledge, have demonstrated efficient excimer emission. In this report, we develop an efficient excimer emitting Pd(ii) complex, *i.e.* Pd3O3 (Fig. 1b), which utilizes a rigid and planar molecular design to achieve efficient blue and white emission while remaining aligned with stable molecular designs. This complex demonstrated efficiencies comparable to its Pt analogs but with higher emission energy and high operational stability. Efficiencies as high as 27.3% were achieved for Pd3O3 devices and one device achieved a device operational lifetime of nearly 3000 h at 1000 cd m^–2^ with an EQE of 19.2% at that same brightness. This performance establishes Pd complexes as an emerging class of emissive materials and demonstrates their potential for stable, efficient, and simplified white OLEDs.

**Fig. 1:**
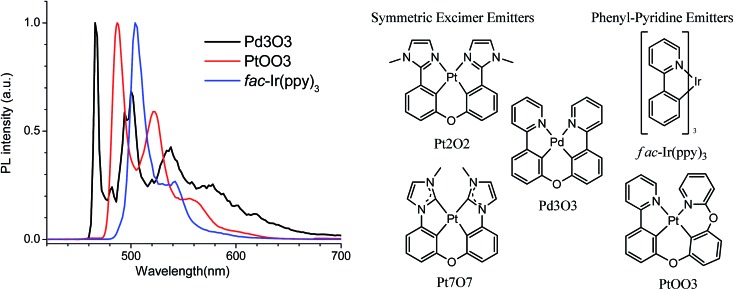
Photoluminescent spectra of Pd3O3, PtOO3, and *fac*-Ir(ppy)_3_ at 77 K in 2-methyl-THF and molecular structures of select tetradentate excimer emitters and phenyl-pyridine based emitters.

## Design of excimer emitters

An efficient and stable Pd complex for single doped white OLEDs requires a rigid molecular structure to suppress the non-radiative pathways that afflict many Pd complexes, a planar molecular geometry to allow sufficient excimer emission, and cyclometalating ligands that are aligned with stable emitter designs. The design of symmetric Pt complexes, such as Pt7O7 and Pt2O2, offer both rigidity and planar geometry necessary for efficient white emission.^38^,^39^ However, these complexes have short operational lifetimes possibly resulting from their phenyl-azole cyclometalating ligands. The designed palladium emitter, Pd3O3, also has a relatively flat planar geometry as shown in the DFT optimized ground state geometry (Fig. S1†) which will make it well suited for excimer emission. Fortunately, Pd3O3, has a higher emission energy than its Pt analogs, allowing high enough triplet energy for warm white emission while using phenyl-pyridine ligands. The comparison of the low temperature emission spectra of Pd3O3 with its Pt and Ir analogs are presented in Fig. 1.^40^ Although the three metal complexes employ the same cyclometalating ligand of phenyl pyridine, the incorporation of palladium metal ions has shifted the maximum emission wavelength of metal complexes from 504 nm for *fac*-Ir(ppy)_3_ to 466 nm for Pd3O3, making Pd3O3 suitable as a phosphorescent emitter for blue and white OLED applications. Moreover, the luminescent lifetime of Pd3O3 at 77 K is around 165 μs, much longer than those of their Ir and Pt analogs in the range of microseconds,^40^,^41^ indicating more localized ligand-centered excited state properties for such Pd complexes.

## Photophysical and electrochemical properties

**Fig. 2:**
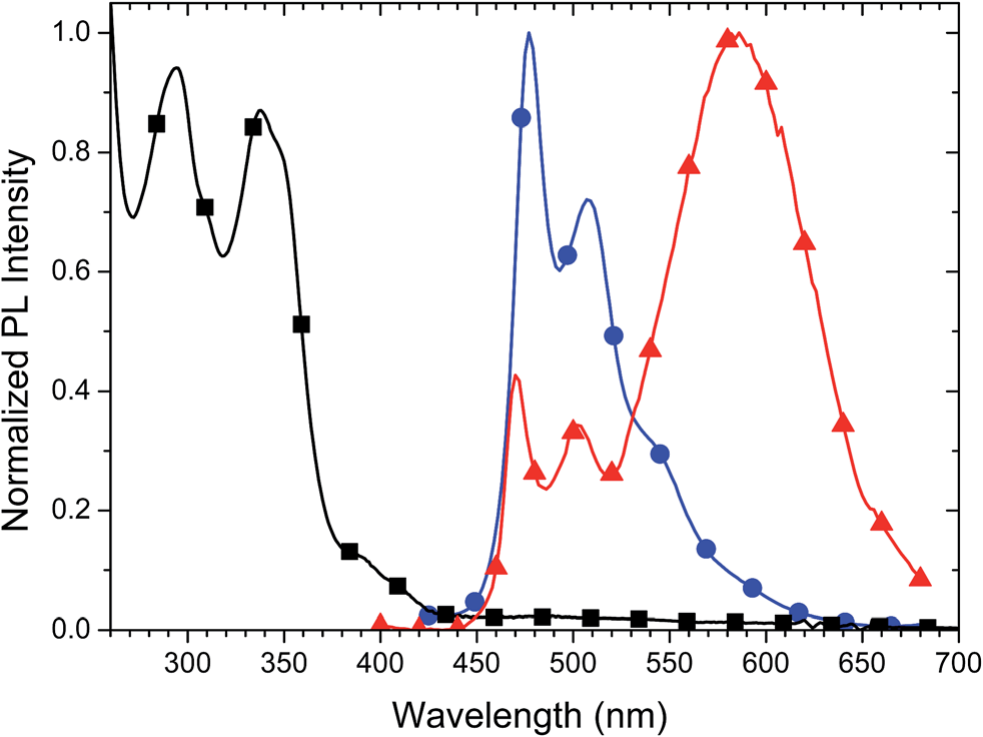
The normalized absorption spectrum of Pd3O3 in DCM (squares) and PL spectrum of a 1% Pd3O3 doped 26mCPy thin film (circles) and PL spectrum of a dilute DCM solution (triangles).

Both a dilute solution of Pd3O3 in dichloromethane (DCM) and a dilute thin film (1% by weight) in 2,6-bis(*N*-carbazolyl)pyridine (26mCPy) were prepared for spectral analysis. The absorption of the solution is shown in Fig. 2. The strong absorption peaks below ∼360 nm are assigned to ^1^π–π* transitions, localized on the phenyl-pyridine ligands. The small shoulder in the 360–450 nm range is assigned to singlet metal to ligand charge transfer (^1^MLCT) transitions similar to previously reported phenyl-pyridine based metal complexes.^40^,^41^ Both the thin film and solution sample show molecular emission peaks in 460–510 nm range. The spectrum of the 1% Pd3O3 doped thin film shows an emission onset near 450 nm with a primary emission peak at 477 nm and a second peak at 507 nm. In contrast, Pd3O3 in a dilute DCM solution contained a broad emission peak at 582 nm even after several dilutions and filtration. This low energy emission band is attributed to excimer emission which is supported by the excitation spectra which showed a shared origin for both the monomer and aggregate emission (Fig. S2†). Furthermore, a PMMA film cast from the same DCM solution shows no excimer emission, confirming the emission was not from ground state aggregates but rather excimer interactions (Fig. S3†). Doped thin films of Pd3O3 in 26mCPy with doping concentrations of 5, 10, and 20 percent Pd3O3 show the expected growth of the excimer emission with increasing concentration (Fig. S4†). The photoluminescent quantum yield (PLQY) of Pd3O3 in a doped PMMA film at room temperature was 87 ± 10% and has a luminescent lifetime of 120 μs indicating that while the radiative rate is low for Pd complexes such as these, the tetradentate complex structure greatly supresses the non-radiative rate, making this emitter a good candidate for efficient OLEDs. The oxidation and reduction potentials were 0.39 V and –2.38 V respectively, determined by differential pulse voltammetry in dimethylformamide *versus* a ferrocene internal reference. These values correspond to a HOMO level of 5.15 eV and a LUMO level of 1.95 eV.^42^,^43^ It is worth noting that these values fall within the redox gap of common host and transport materials such as mCBP or mCPy and is likely to improve charge and exciton confinement.^32^,^42^,^43^

## White OLED device performance

**Fig. 3:**
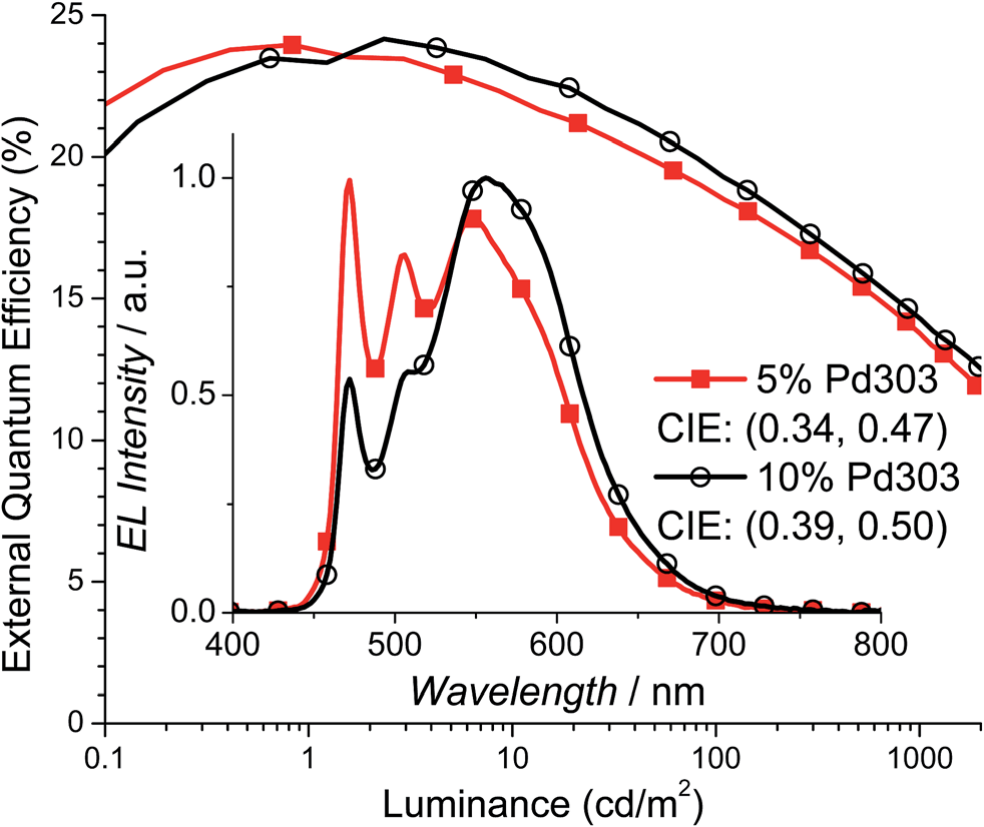
External quantum efficiency *versus* luminance and electroluminescent spectra (inset) for Pd3O3 devices with 5% (squares) and 10% (circles) dopant concentrations in the structure: ITO/HATCN/NPD/TAPC/*x*% Pd3O3:26mCPy/DPPS/BmPyPB/LiF/Al.

To evaluate the performance of Pd3O3 in a WOLED setting, devices were fabricated in a known efficient and charge confining structure: ITO/HATCN (10 nm)/NPD (40 nm)/TAPC (10 nm)/*x*% Pd3O3:26mCPy (25 nm)/DPPS (10 nm)/BmPyPB (40 nm)/LiF/Al for dopant concentrations of 5% and 10% Pd3O3 by mass.^44^–^46^ HATCN is 1,4,5,8,9,11-hexaazatriphenylene-hexacarbonitrile, NPD is *N*,*N*′-diphenyl-*N*,*N*′-bis(1-naphthyl)-1,1′-biphenyl-4,4′′-diamine, TAPC is di-[4-(*N*,*N*-di-tolyl-amino)-phenyl]cyclohexane, 26mCPy is 2,6-bis(*N*-carbazolyl)pyridine, DPPS is diphenyl-bis[4-(pyridin-3-yl)phenyl]silane, and BmPyPB is 1,3-bis[3,5-di(pyridin-3-yl)phenyl]benzene. As shown in Fig. 3, Pd3O3 devices at 5% and 10% dopant concentration show high peak EQEs of 23.9% and 24.2% respectively. This is the highest reported efficiencies for OLEDs employing Pd complexes regardless of emission color.^33^–^35^ The efficiency drops to 18.5% and 13.8% at 100 cd m^–2^ and 1000 cd m^–2^ for the 5% doped device and 19.3% and 14.3% at 100 cd m^–2^ and 1000 cd m^–2^ for the 10% doped device. This device efficiency roll-off is likely due to the combination of poor charge balance at high current density and the long PL emission lifetime of 120 μs.^47^ The peak power efficiencies (PE) were also high for both these devices with 65.3 Lm per W and 67.9 Lm per W (Fig. S5†) for the 5% and 10% doped devices, albeit with non-ideal color coordinates of (0.34, 0.47) and (0.39, 0.50), respectively.

The emission spectra in the inset to Fig. 3 shows a monomer emission peak at 472 nm and a broad excimer peak at 550–600 nm. At the dopant concentration of 5%, the excimer peak and monomer peak are approximately equal heights yielding warm white light with CIE coordinates of (0.34, 0.47) and CRI of 53. When the concentration of Pd3O3 is increased to 10%, the excimer emission broadens and increases to approximately twice the height of the monomer emission. Consequently, the emission is orange with CIE coordinates of (0.39, 0.50) and a CRI of 52. It should also be noted that the monomer to excimer emission balance occurs at a much lower dopant concentration than many of the reported platinum complexes, yielding an emission spectrum with non-ideal CIE coordinates.^38^,^39^ This is due to the preferential stacking of Pd3O3 molecules which was also reflected in the poor solubility of Pd3O3.^48^,^49^ Furthermore, the excimer emission drops off rapidly at 600 nm missing a significant portion of the red spectrum leading in part to the low CRI. Modifying the planar geometry nature of Pd3O3 molecules by adding steric substitutional groups or using bulky bridging ligands will allow stronger molecular interaction between emissive materials and the host molecules and can tune the monomer and excimer emission colors to yield more ideal white color.^50^,^51^

## Devices with long operational lifetimes

Currently, there are very few reports on the operational lifetime of white OLED devices and even less is known about the operational stability of excimer based WOLEDs. Due to the known instability of the TAPC and DPPS blocking materials, separate Pd3O3 devices were fabricated in stable device structure: ITO/HATCN (10 nm)/NPD (40 nm)/TrisPCz (10 nm)/EML/BAlq (10 nm)/BPyTP (40 nm)/LiF (1 nm)/Al. The EML is varied as follows:

Device 1: 6% Pd3O3: 26mCPy (30 nm).

Device 2: 10% Pd3O3: 26mCPy (10 nm)/6% Pd3O3: 26mCPy (20 nm).

Device 3: 20% Pd3O3: 26mCPy (10 nm)/6% Pd3O3: 26mCPy (20 nm).

**Fig. 4:**
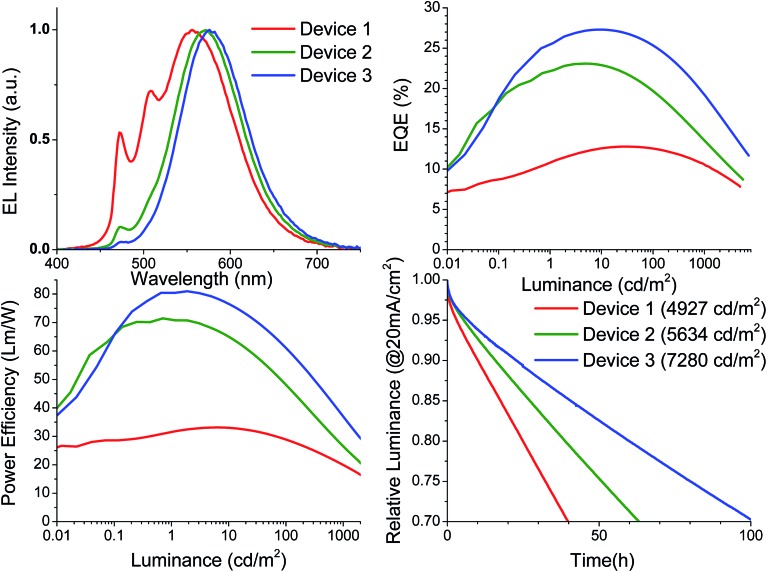
(a) Electroluminescent spectra, (b)external quantum efficiency *versus* luminance, (c) power efficiency *versus* luminance and (d) operational lifetime for Pd3O3 in Device 1 (red), Device 2 (green), and Device 3 (blue). The device operational lifetime was measured at a constant drive current of 20 mA cm^–2^.

Where TrisPCz is 9,9′,9′′-triphenyl-9H,9′H,9′′H-3,3′:6′3′′-tercarbazole, BAlq is bis(2-methyl-8-quinolinolato)(biphenyl-4-olato)aluminum, and BPyTP is 2,7-di(2,2′-bipyridin-5-yl)triphenylene, which are selected based on the previous literature reports related to stable fluorescent and phosphorescent devices.^52^,^53^ Devices 1 was fabricated with a fixed dopant concentration 6%. As seen in Fig. 4a, the resulting spectrum for Device 1 contains a dominant excimer peak as well as a monomer peak at 472 nm that is approximately 50% of the excimer peak height. The monomer is suppressed compared to that of the 5% doped device in the charge confining structure despite their similar emissive layers. The reason for this reduction may be related to charge balance, morphological differences, or quenching of the monomeric emission by the TrisPCz blocking layers. Significant NPD emission is observed upon removal of the TrisPCz blocking layer in a similar device structure (Fig. S6†) suggesting significant exciton density near the EML/HTL interface. Despite this spectral change, the 6% doped device still achieved warm white emission with CIE coordinates of (0.37, 0.51) but a poor CRI of 48. Device 1 achieved a peak EQE of 12.8% and a peak power efficiency of 33.1 Lm per W, both of which dropped to 10.7% and 19.9 Lm per W, respectively, at 1000 cd m^–2^. The device operational lifetime was measured at accelerated conditions of a constant current of 20 mA cm^–2^, which was summarized in Table 1. A nominal lifetime to 70% initial luminance (LT_70_) for Device 1 was measured to be 40 hours at an initial luminance of 4927 cd m^–2^. This corresponds to roughly 600 hours at a practical luminance of 1000 cd m^–2^, using the formula LT(*L*_1_) = LT(*L*_0_)(*L*_0_/*L*_1_)^*n*^, where *L*_1_ is the desired luminance and the exponent *n* is assumed to be a moderate value of 1.7.^54^

**Table 1:** Summary of device performance for stable devices of Pd3O3

Device	CRI	CIE	EQE (%)		PE (Lm per W)		LT_70_		
			Peak	1000 cd m^–2^	Peak	1000 cd m^–2^	*L*_0_ (cd m^–2^)	@*L*_0_	@1000 cd m^–2^
1	48	(0.37, 0.51)	12.8	10.7	33.1	19.9	4927	40	601
2	45	(0.45, 0.51)	23.1	13.6	71.4	26.5	5634	63	1190
3	43	(0.48, 0.50)	27.3	19.2	81.0	36.9	7280	101	2950

Previous reports have shown that while the use of TrisPCz improves device efficiency due to its charge blocking capabilities, the devices also suffers a loss in nominal operational lifetime. Such an effect is also observed for Pd3O3 devices, and could be due to possible charge build-up at the interface of EML/EBL or due to inherent instabilities of the TrisPCz layer.^47^,^52^,^53^ In either case both high operational lifetimes and high efficiencies may be achieved by shifting the charge recombination zone toward the center of the emissive layer. Charge build-up near the TrisPCz interface can be explained by the energy level alignment of Pd3O3 in the 26mCPy host. The LUMO level of Pd3O3 (1.95 eV) is a shallow trap in 26mCPy (1.6 eV) whereas the HOMO level of Pd3O3 (5.15 eV) is a much deeper trap in 26mCPy (6.0 eV). This would likely lead to an imbalance in charge transport due to the higher energetic barriers for the successive hopping of holes in and out of the traps. To compensate for the imbalance the doping concentration near the electron blocking layer was increased to 10% for Device 2 and 20% for Device 3. Increasing the doping concentration expectedly increased the excimer contribution to the emission ultimately leading to a shift in color from white to orange with CIE coordinates of (0.48, 0.50) for Device 3. Both the EQE and power efficiencies of the devices were improved dramatically yielding a peak EQE of 27.3% and a power efficiency of 81 Lm per W for Device 3. Remarkably, at 1000 cd m^–2^ the EQE remained at 19.2% and the power efficiency at 36.9 Lm per W despite the long phosphorescent lifetime of 120 μs. Accelerated operational lifetime testing was carried out on Device 2 and 3, exhibiting improved nominal operational lifetimes at 20 mA cm^–2^ compared to Device 1. The use of a 10% doped layer for Device 2 achieved an LT_70_ of 62 hours at 5634 cd m^–2^ corresponding to 1190 hours at 1000 cd m^–2^, while the use of a 20% doped layer further improved the operational lifetime to an LT_70_ of 101 hours at 7280 cd m^–2^ corresponding to 2950 hours at 1000 cd m^–2^. The higher efficiencies, low roll-off, and extended operational lifetimes all suggest a significant improvement in charge balance inside the device.^47^ The ability to simultaneously achieve long lifetime and high efficiency in the same device represents a major step forward in the development of stable single doped white OLEDs. The nearly 20% EQE and LT_70_ of 3000 hours at 1000 cd m^–2^ push both palladium complexes and their use in excimer based white OLEDs to the forefront of academic efforts in OLED development. Furthermore, with incorporation of light outcoupling techniques, doubling the luminance at a given driving condition could be reasonably expected to yield lifetimes of 10 000 hours at 1000 cd m^–2^ which approaches the minimum commercialization requirement.^54^ Our progress represents a substantial improvement upon previous reports of Pt-based excimers which demonstrated less than 100 h for Pt7O7 or 400 h for Pt1O2me_2_ at 1000 cd m^–2^, both of which were only in the range of 8–12% efficient.^38^,^39^ This indicates the importance of maximizing emissive material performance through judicious molecular design and rational device architecture optimization.

## Conclusions

In summary, an efficient and stable blue emitting palladium complex, Pd3O3, was designed and prepared, enabling efficient and stable single-doped WOLED for lighting applications. Devices employing this complex achieved peak efficiencies over 27% which are comparable to the state of the art Ir and Pt analogs in a similar device structure, demonstrating the value of this new class of complexes.^10^–^25^ Furthermore, the ability to achieve higher energy emission for a given cyclometalating ligand makes this class of complex particular interesting for both blue and white OLED applications. One stable excimer emitting device achieved a peak efficiency 27.3% and 81 Lm per W while also having a power efficiency of 36.9 Lm per W and operational lifetime of nearly 3000 h at 1000 cd m^–2^. Further development of the emissive materials and fine tuning of the emissive layer is expected to improve the color quality to achieve efficient and stable single doped white OLEDs. Thus, the introduction of efficient and stable palladium complexes and their use in single doped white OLEDs could provide a route for efficient low-cost solid state lighting. While this particular complex rapidly formed excimers even at low doping concentrations, this material and corresponding device design demonstrates the potential for simultaneously achieving high efficiencies and long operational lifetimes in devices with high triplet energy emitters. It is worth noting that Pd3O3 demonstrates the longest device operational lifetime in the academic literature for any phosphorescent emitter with triplet energy of over 2.6 eV.^29^,^32^ The knowledge and experience gained from these results will open a new avenue of developing stable and efficient blue phosphorescent emitters for display and lighting applications.

## Experimental

### General synthetic procedure

All commercial reagents were purchased and used as received without further purification. Pd(OAc)_2_ was purchased from Pressure Chemical Co. *n*-Bu_4_NBr, CuI, 2-(tributylstannyl)pyridine and 2-picolinic acid were purchased from Sigma Aldrich. Silica gel (40–60 μm) was purchased from Agela Technologies and BDH. Solvents DMSO, toluene (low water), acetate acid were purchased from Alfa Aesar, J. T. Baker, Fluke and BDH respectively. All reactions were carried out under an inert N_2_ atmosphere in oven-dried glassware. External bath temperatures were used to record all reaction temperatures. Flash column chromatography was carried out with silica gel. Proton and carbon NMR spectra (^1^H NMR and ^13^C NMR) were recorded in dimethyl sulfoxide-*d*_6_ (DMSO-*d*_6_) on a Varian 400 MHz NMR spectrometer. The solvent residual peak (DMSO-*d*_6_) was calibrated to 2.50 ppm for ^1^H NMR and 39.52 ppm for ^13^C NMR. Multiplicities are abbreviated as follows: s = singlet, d = doublet, t = triplet, br = broad, m = multiplet.

### The synthesis of Pd3O3

2-(3-(3-(Pyridin-2-yl)phenoxy)phenyl)pyridine (470 mg, 1.45 mmol), Pd(OAc)_2_ (348 mg, 1.55 mmol), and *n*-Bu_4_NBr (48 mg, 0.149 mmol) were added into a 100 ml three-neck round-bottom flask, then 30 ml acetic acid was added. The mixture was bubbled with nitrogen for 30 minutes then stirred at ambient temperature for 12 hours. The mixture was heated in an oil bath at a temperature of 110 °C for another 72 hours. 100 ml of water was added after the mixture was cooled down to room temperature. The precipitate was collected through filtration, washed with water for three times then dried in air. The collected solid was purified through column chromatography on silica gel using dichloromethane as eluent to afford the desired palladium complex Pd3O3 as a light yellow solid 390 mg in 63% yield. ^1^H NMR (DMSO-*d*_6_, 400 MHz): *δ* 7.16 (d, *J* = 7.6 Hz, 2H), 7.27 (t, *J* = 8.0 Hz, 2H), 7.55 (t, *J* = 6.4 Hz, 2H), 7.74 (d, *J* = 7.2 Hz 2H), 8.09–8.15 (m, 2H), 8.28 (d, *J* = 8.4 Hz, 2H), 8.96 (d, *J* = 5.2 Hz, 2H). MS (APCI^+^) *m*/*z*: [M]^+^ calcd for C_22_H_15_0N_2_OPd 429.0219, found 429.0232. Anal. calcd for C_22_H_14_N_2_OPd: C, 61.63; H, 3.29; N, 6.53; found: C, 61.70; H, 3.31; N, 6.62.

### Materials

TAPC (di-[4-(*N*,*N*-di-tolyl-amino)-phenyl]cyclohexane),^46^ TrisPCz (9,9′,9′′-triphenyl-9H,9′H,9′′H-3,3′:6′3′′-tercarbazole),^53^ 26mCPy (2,6-bis(*N*-carbazolyl)pyridine),^49^ DPPS (diphenyl-bis[4-(pyridin-3-yl)phenyl]silane),^44^ BmPyPB (1,3-bis[3,5-di(pyridin-3-yl)phenyl]benzene),^45^ and BPyTP (2,7-di(2,2′-bipyridin-5-yl)triphenylene)^53^ were all synthesized following previous literature reports. HATCN (1,4,5,8,9,11-hexaazatriphenylene-hexacarbonitrile), NPD (*N*,*N*′-diphenyl-*N*,*N*′-bis(1-naphthyl)-1,1′-biphenyl-4,4′′-diamine), BAlq (bis(2-methyl-8-quinolinolato)(biphenyl-4-olato) aluminum), and mCBP (3,3-di(9*H*-carbazol-9-yl) biphenyl) were all provided from commercial suppliers. All materials were sublimed in a 4-zone thermal gradient furnace at pressures of 10^–5^ torr prior to use.

### Materials characterization

The photoluminescent spectra were measured on a Horiba Jobin Yvon FluoroLog-3 spectrometer. The absorption spectrum was measured on an Agilent 8453 UV-visible spectrometer. Doped thin films for photoluminescent quantum yield (PLQY) measurements were fabricated by the drop-cast process of Pd3O3 in a PMMA host. The PLQY measurements were carried out on a Hamamatsu absolute PL quantum yield spectrometer model C11347. It should be noted that the error bar may be large for the PLQY measurement with emissive materials which have long lifetimes (>100 μs) due to quenching by residual oxygen in the integration sphere. Cyclic voltammetry and differential pulsed voltammetry were performed on a CHI610B electrochemical analyzer in a solution of anhydrous DMF using 0.1 M tetra(*n*butyl) ammonium hexafluorophosphate as the supporting electrolyte. A ferrocene/ferrocenium (Fc/Fc^+^) redox couple used as an internal reference. The HOMO and LUMO values were determined following literature reported fits relating the electrochemical potentials to the values determined *via* ultraviolet photoemission spectroscopy and inverse photoelectron spectroscopy, respectively.^42^,^43^ Mass spectra were recorded on JEOL GCmate gas chromatograph/mass spectrometer. Elemental analysis was carried out with a Perkin-Elmer 2400 CHN Elemental Analyzer.

### Device fabrication and characterization

Devices were fabricated on pre-patterned substrates of ITO on glass purchased from Optics Balzers (50 Ω per sq.). Prior to deposition substrates were cleaned by a gentle scrub followed by subsequent sonication in water, acetone, and isopropanol. Organic layers were deposited by vacuum thermal evaporation in a custom made chamber by Travato Man. Inc. Base pressures were kept between 10^–8^ to 10^–7^ torr and deposition rates were kept between 0.5–1.0 Å s^–1^. A 1 nm LiF buffer layer was deposited at 0.2 Å s^–1^. Al cathodes were deposited without breaking vacuum at 1–2 Å s^–1^ through a shadow mask defining a device area of 4 mm^2^.

High efficiency devices were fabricated in the structure: ITO/HATCN (10 nm)/NPD (40 nm)/TAPC(10 nm)/*x*% Pd3O3:26mCPy (25 nm)/DPPS (10 nm)/BmPyPB (40 nm)/LiF/Al where *x* = 5% or 10%. For stable devices the following structure was used: ITO/HATCN (10 nm)/NPD (40 nm)/TrisPCz (10 nm)/EML/BAlq (10 nm)/BPyTP (40 nm)/LiF (1 nm)/Al. The EML is varied as follows:

Device 1: 6% Pd3O3: 26mCPy (30 nm).

Device 2: 10% Pd3O3: 26mCPy (10 nm)/6% Pd3O3: 26mCPy (20 nm).

Device 3: 20% Pd3O3: 26mCPy (10 nm)/6% Pd3O3: 26mCPy (20 nm).

Current–voltage–luminance characteristics were taken with a Keithley 2400 Source-Meter and a Newport 818 Si photodiode inside a nitrogen-filled glove-box with all devices assumed to be Lambertian emitters. Accelerated lifetime testing was performed in a nitrogen filled glove box without encapsulation at a constant current of 20 mA cm^–2^. Extrapolated lifetimes were approximated using the formula LT(*L*_1_) = LT(*L*_0_)(*L*_0_/*L*_1_)^1.7^.^54^ EL spectra were taken at 1 mA cm^–2^ using a calibrated ocean optics USB 2000 spectrometer.

## Conflicts of interest

There are no conflicts of interest to declare.

## Supplementary Material

Supplementary information
