# Supplementary material for: Efficient and stable single-doped white OLEDs using a palladium-based phosphorescent excimer
Source: Chem Sci. 2017 Sep 11;8(12):7983–90. doi: 10.1039/c7sc02512b (PMC5853771; doi:10.1039/c7sc02512b)
Supplement: Supplementary file 1 [file SC-008-C7SC02512B-s001.pdf]

Electronic Supplementary Material (ESI) for Chemical Science.
This journal is © The Royal Society of Chemistry 2017

Supporting Information

Efficient and Stable Single-Doped White OLEDs Using a Palladium-Based Excimer

Tyler Fleetham, Yunlong Ji, Liang Huang, Trenten Fleetham, and Jian Li*
Materials Science and Engineering, Arizona State University, Tempe, AZ, 85287, USA

Synthesis of Materials

General Experimental Details.

All commercial reagents were purchased and used as received without further purification. $Pd(OAc)_2$ was purchased from Pressure Chemical Co. *n*-Bu_4NBr, CuI, 2-(tributylstannyl)pyridine and 2-picolinic acid were purchased from Sigma Aldrich. Silica gel (40-60μm) was purchased from Agela Technologies and BDH. Solvents DMSO, toluene (low water), acetate acid were purchased from Alfa Aesar, J. T. Baker, Fluke and BDH respectively.

All reactions were carried out under an inert N_2 atmosphere in oven-dried glassware. External bath temperatures were used to record all reaction temperatures. Flash column chromatography was carried out with silica gel. Proton and carbon NMR spectra (^{1}H NMR and ^{13}C NMR) were recorded in Dimethyl Sulfoxide-d_6 (DMSO-d_6) on a Varian 400 MHz NMR spectrometer. The solvent residual peak (DMSO-d_6) was calibrated to 2.50 ppm for ^{1}H NMR. Multiplicities are abbreviated as follows: s = singlet, d = doublet, t = triplet, br = broad, m = multiplet.

Synthesis of 2-(3-(3-(pyridin-2-yl)phenoxy)phenyl)pyridine

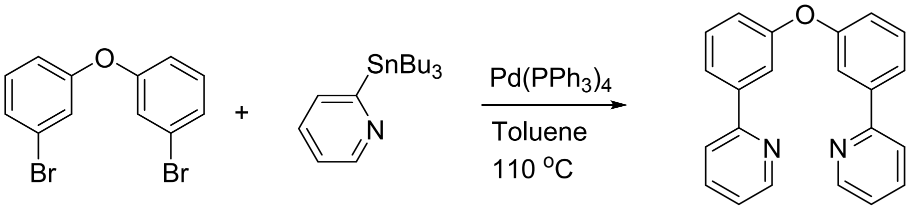

To a 100 mL three-neck round-bottom flask were added 1-bromo-3-(3-bromophenoxy)benzene (656 mg, 2 mmol) and 2-(tributylstannyl)pyridine (1.76 g, 4.8 mmol), the flask was evacuated and backfilled with nitrogen for three times, Tetrakis(triphenylphosphine)palladium(0) (115 mg, 0.1 mmol) and toluene (20 mL) were added under the protection of nitrogen, the reaction mixture was stirred at 110 °C under nitrogen for 24 hours. After cooling to room temperature, the mixture was poured into 50 mL of water and extracted with ethyl acetate (100 mL*3), the combined organic layer was dried with anhydrous Na_2SO_4 and concentrated under reduced pressure. Purification by column chromatography (hexane: ethyl acetate=5:1) afford the desired product as white solid (550 mg, 84%) ^{1}H NMR (DMSO-d_6, 400 MHz): 7.16 (dd, J = 8.0, 2.4 Hz, 2 H), 7.33-7.38 (m, 2 H), 7.54 (t, J = 7.6 Hz, 2 H), 7.79 (m, 2 H), 7.85-7.91(m, 4 H), 7.98(d, J = 8 Hz, 2 H), 8.63(d, J = 4.4 Hz, 2 H)

Synthesis of Palladium (II) 2-(3-(3-(pyridin-2-yl)phenoxy)phenyl)pyridine.

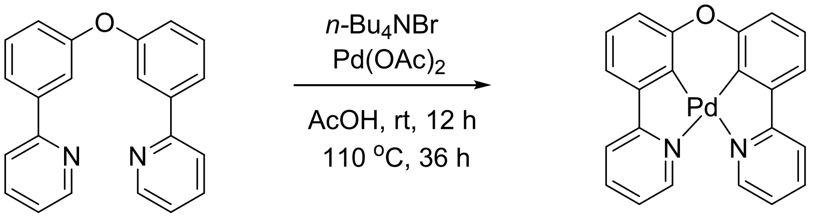

2-(3-(3-(pyridin-2-yl)phenoxy)phenyl)pyridine (470 mg, 1.45 mmol), $Pd(OAc)_2$ (348 mg, 1.55 mmol), and n-Bu_4NBr(48 mg, 0.149 mmol) were added into a 100 ml three-neck round-bottom flask, then 30 ml acetic acid was added. The mixture was bubbled with nitrogen for

30 minutes then stirred at ambient temperature for 12 hours. The mixture was heated in an oil bath at a temperature of 110 °C for another 72 hours. 100 ml of water was added after the mixture was cooled down to room temperature. The precipitate was collected through filtration, washed with water for three times then dried in air. The collected solid was purified through column chromatography on silica gel using dichloromethane as eluent to afford the desired Palladium complex Pd3O3 as a light yellow solid 390 mg in 63% yield. ^{1}H NMR (DMSO-d_6, 400 MHz): δ 7.16 (d, J = 7.6 Hz, 2 H), 7.27 (t, J = 8.0 Hz, 2 H), 7.55 (t, J = 6.4 Hz, 2 H), 7.74 (d, J = 7.2 Hz 2 H), 8.09-8.15 (m, 2 H), 8.28 (d, J = 8.4 Hz, 2 H), 8.96(d, J = 5.2 Hz, 2 H). MS (APCI+) m/z: [M]+ Calcd for $C_{22}H_{15}0N_2OPd$ 429.0219, Found 429.0232. Anal. Calcd. For $C_{22}H_{14}N_2OPd$: C, 61.63; H, 3.29; N, 6.53; Found: C, 61.70; H, 3.31; N, 6.62.

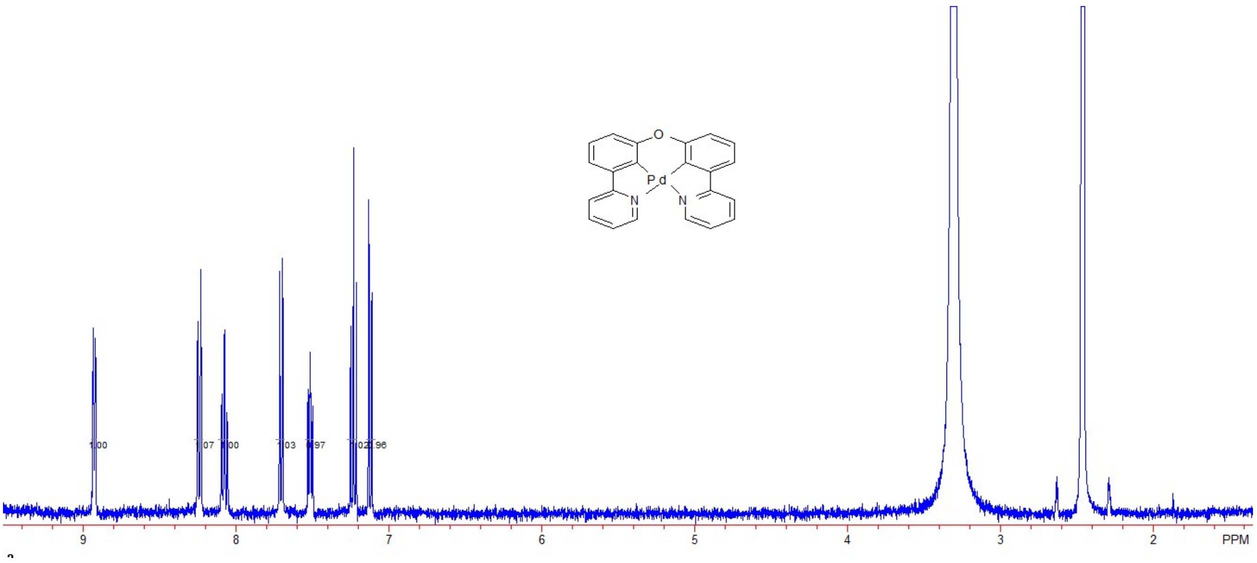

^{1}H NMR of Pd303 (400 MHz, DMSO-d_6)

Device Experimental Details.

Materials: TAPC (di-[4-(N,N-di-toylyl-amino)-phyenyl]cyclohexane),[1] TrisPCz (9,9′,9″-triphenyl-9H,9′H,9″H-3,3′:6′3″-tercarbazole),[2] 26mCPy (2,6-bis(N-carbazolyl) pyridine),[3] DPPS (diphenyl-bis[4-(pyridin-3-yl)phenyl]silane),[4] BmPyPB (1,3-bis[3, 5-di(pyridin-3-yl)phenyl]benzene),[5] and BPyTP (2,7-di(2,2′-bipyridin-5-yl)triphenylene)[2] were all synthesized following previous literature reports. HATCN (1,4,5,8,9,11-hexaazatriphenylene-hexacarbonitrile), NPD (N,N'-diphyenyl-N,N'-bis(1-naphthyl)-1,1'-biphenyl-4,4"-diamine), BAlq bis(2-methyl-8-quinolinolato)(biphenyl-4-olato)aluminum, and mCBP 3,3-di(9*H*-carbazol-9-yl)biphenyl were all provided from commercial suppliers. All materials were sublimed 1 or more times in a 4-zone thermal gradient furnace at pressures of 10^{-5} torr prior to use.

Device Fabrication and Characterization: Devices were fabricated on pre-patterned substrates of ITO on glass. Prior to deposition substrates were cleaned by a gentle scrub followed by subsequent sonication in water, acetone, and isopropanol. Organic layers were deposited by vacuum thermal evaporation in a custom made chamber by Travato Man. Inc. Base pressures were kept between 10^{-8} -10^{-7} torr and deposition rates were kept between 0.5-1.0 Å/s. A 1nm LiF buffer layer was deposited at 0.2 Å/s. Al cathodes were deposited without breaking vacuum at 1-2 Å/s through a shadow mask defining a device area of 4 mm^2.

High efficiency devices were fabricated in the structure: ITO/HATCN (10 nm)/NPD (40 nm)/ TAPC(10 nm)/x% Pt3O3: 26mCPy (25 nm) /DPPS (10nm)/BmPyPB (40 nm)/LiF/Al where x=5% or 10%. For stable devices the following structure was used: ITO/HATCN(10 nm)/ NPD(40 nm)/TrisPCz (0 or 10 nm)/x% Pd3O3:Host(25 nm)/BAlq(10 nm)/BPyTP(40 nm)/LiF/Al where x=2% or 10% and the host is either 2,6 mCPy or mCBP. Current-voltage-luminance characteristics were taken with a Keithley 2400 Source-Meter and a Newport 818 Si photodiode inside a nitrogen-filled glove-box with all devices assumed to be Lambertian

emitters. Accelerated lifetime testing was performed at a constant current of 20 mA/cm^2. EL spectra were taken at 1mA/cm^2 using a calibrated ocean optics HR4000 spectrometer.

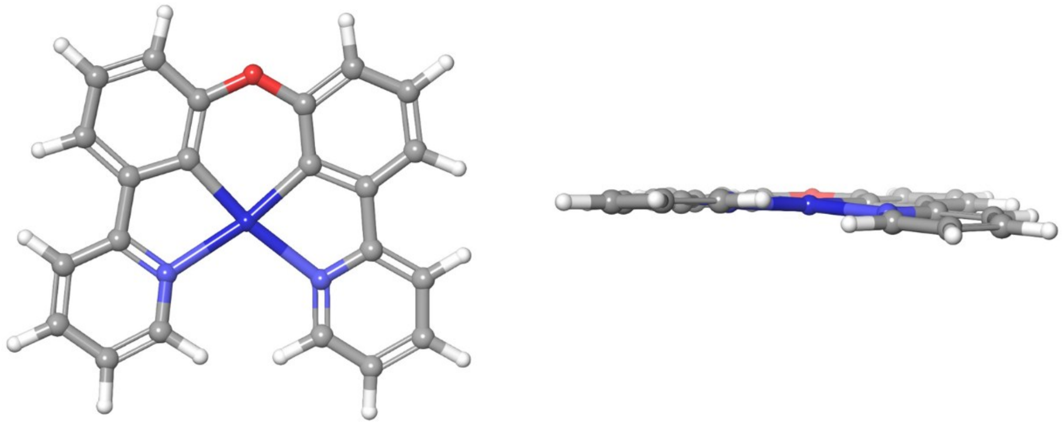

Figure S1: Density functional theory optimized ground state geometry of Pd3O3 using B3LYP functional and LACVP** basis set.

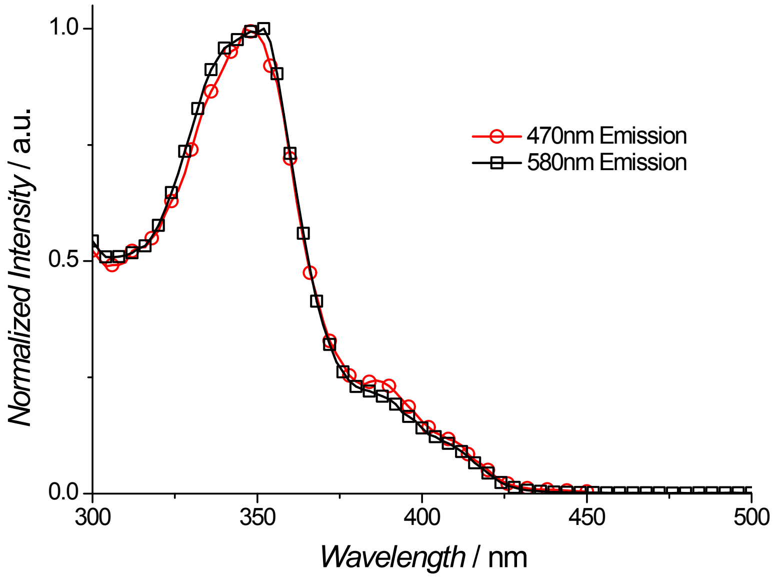

Figure S2: Excitation spectra for a solution of Pd3O3 in dichloromethane with emission measured at 470 nm (circles) and 580 nm (squares).

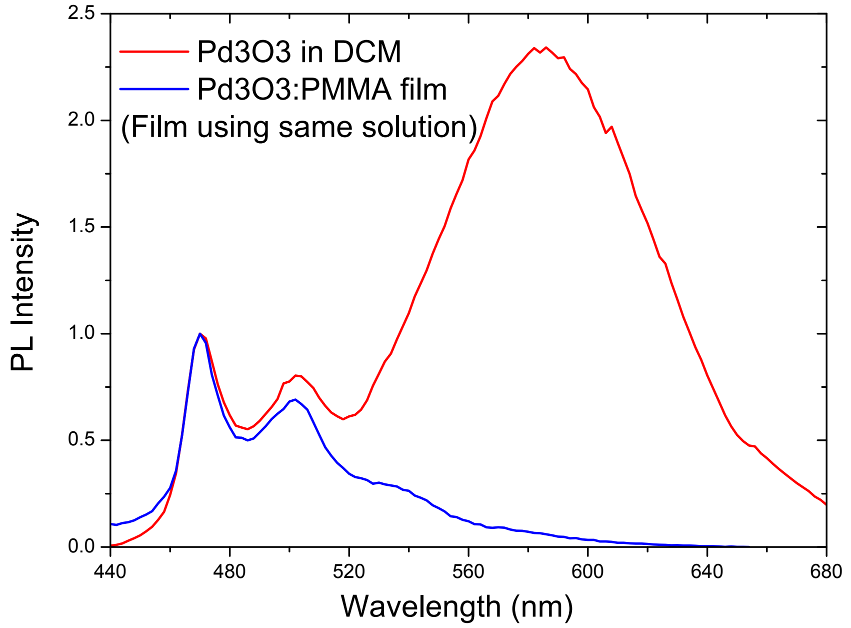

Figure S3. PL spectra of Pd3O3 in a solution of DCM (Red) and doped in PMMA (blue). PMMA was dissolved in the dilute solution after the solution PL spectra was collected and the solution was spun cast on a quartz substrate. The dissappearance of the excimer peak indicate that the emission is not due to aggregates or dimers.

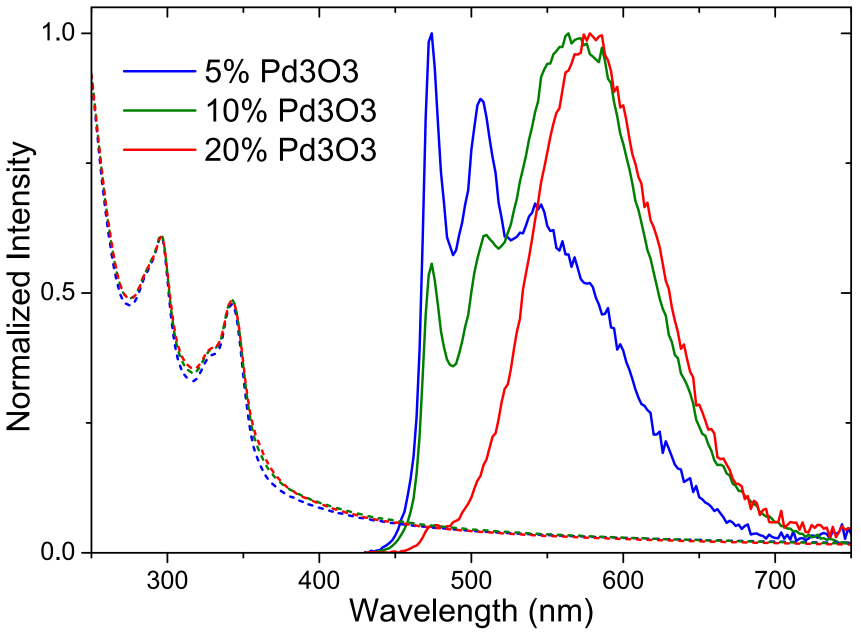

Figure S4: Normalized absoprtion spectra (dashed) and emission spectra (solid) from Pd3O3 doped PMMA films.

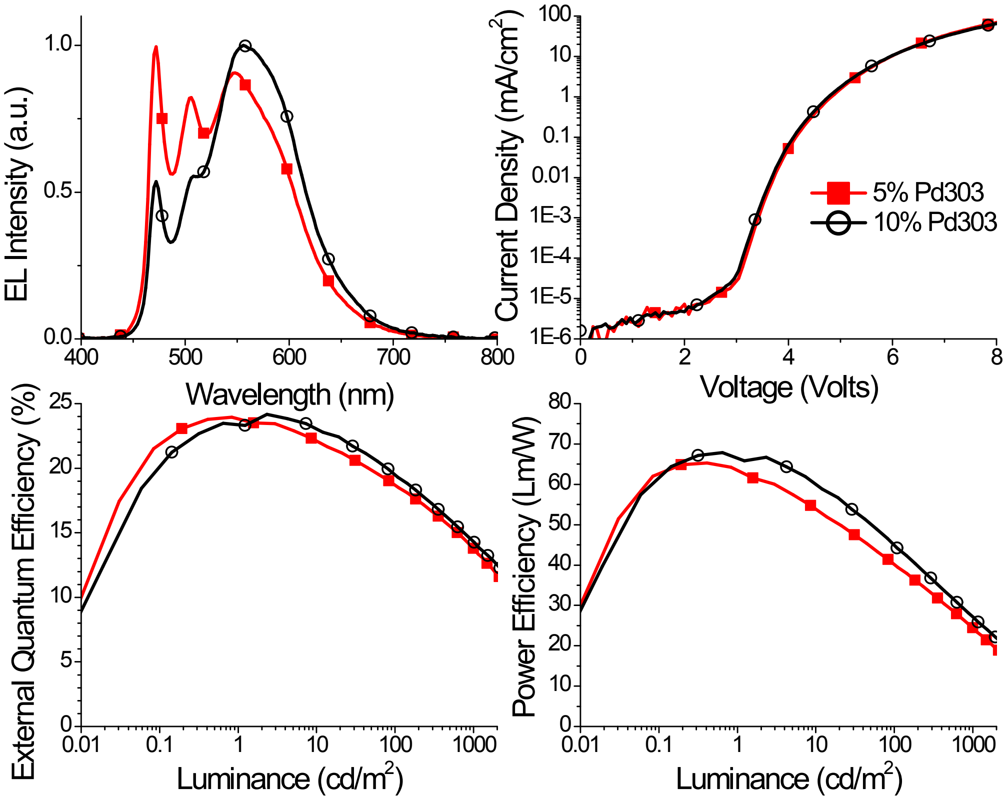

Figure S5: a) Electroluminescent spectra, b) current density-voltage characteristics, c) external quantum efficiency, and d) power efficiency of Pd3O3 devices in the structure: ITO/HATCN (10 nm)/NPD (40 nm)/ TAPC(10 nm)/x% Pt3O3: 26mCPy (25 nm) /DPPS (10 nm)/BmPyPB (40 nm)/LiF/Al for Pt3O3 concentrations of 5% (squares) and 10% (circles).
Device S1: ITO/HATCN/ NPD/TrisPCz /10% Pd3O3:mCBP/BAlq/BPyTP/LiF/Al
Device S2: ITO/HATCN/ NPD/10% Pd3O3:mCBP/BAlq/BPyTP/LiF/Al
Device S3: ITO/HATCN/ NPD/TrisPCz /10% Pd3O3: mCBP /BAlq/BPyTP/LiF/Al
Device S4: ITO/HATCN/ NPD/10% Pd3O3: mCBP/BAlq/BPyTP/LiF/Al

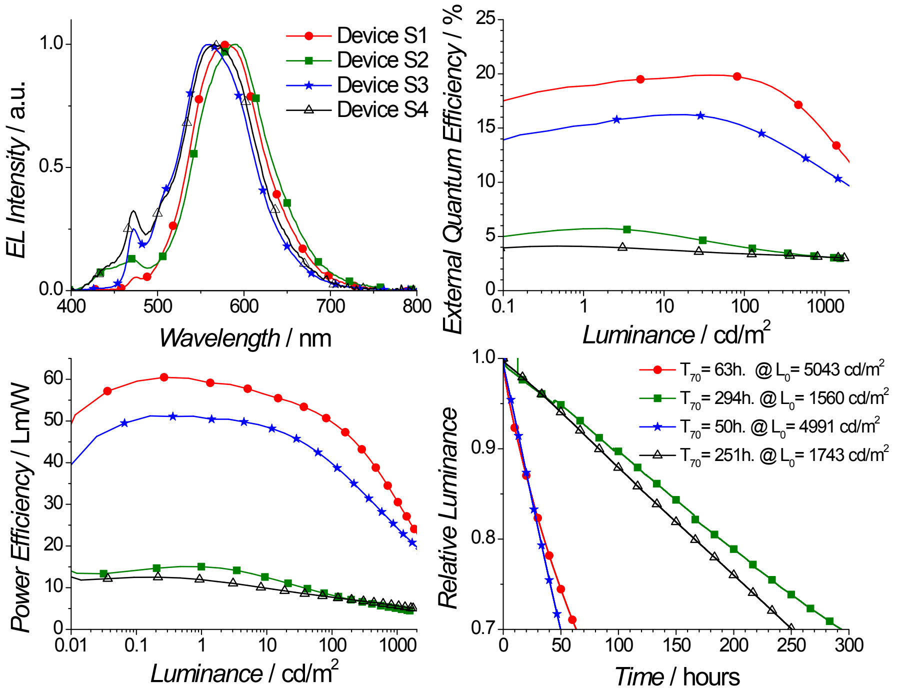

Figure S6. a)Electroluminescent spectra, b)external quantum efficiency versus luminance, c) power efficiency versus luminance and d) operational lifetime for Pd3O3 in Device S1 (circles), Device S2 (squares), Device S3 (stars), and Device S4 (triangles). The device operational lifetime was measured at a constant drive current of 20 mA/cm^2.

(1) N Chopra, N. Swensen, J. S.; Polikarpov, E.; Cosimbescu, L.; So, F. Padmaperuma, A. B. High efficiency and low roll-off blue phosphorescent organic light-emitting devices using mixed host architecture. *Appl. Phys. Lett.* **2010**, *97*, 033304;

(2) Nakanotani, H.; Masui, K.; Nishide, J.; Shibata, T.; Adachi, C. Promising operational stability of high-efficiency organic light-emitting diodes based on thermally activated delayed fluorescence. *Sci. Rep.* **2013**, *3*, 2127.

(3) Williams, E.L.; Haavisto, K.; Li, J.; Jabbour, G.E. Excimer-Based White Phosphorescent Organic Light-Emitting Diodes with Nearly 100 % Internal Quantum Efficiency. *Adv. Mater.* **2007**, *19*, 197-202

(4) Xiao, L.; Su, S-J.; Agata, Y.; Lan, H.; Kido, J. Nearly 100% Internal Quantum Efficiency in an Organic Blue-Light Electrophosphorescent Device Using a Weak Electron Transporting Material with a Wide Energy Gap. *Adv. Mater.* **2009**, *21*, 1271 –1274;

(5) Sasabe, H. ; Gonmori, E. ; Chiba, T. ; Li, Y.J. ; Tanaka, D.; Su, S.-J. ; Takeda, T. ; Pu, Y-J.; Nakayama, K.; Kido, J. Wide-Energy-Gap Electron-Transport Materials Containing 3,5-Dipyridylphenyl Moieties for an Ultra High Efficiency Blue Organic Light-Emitting Device. *Chem. Mater.* **2008**, *20*, 5951 –5953;
